# Supplementary material for: Inflammation is a critical factor for successful regeneration of the adult zebrafish retina in response to diffuse light lesion
Source: Front Cell Dev Biol. 2024 Jul 12;12:1332347. doi: 10.3389/fcell.2024.1332347 (PMC11272569; doi:10.3389/fcell.2024.1332347)
Supplement: Supplementary file 1 [file DataSheet1.docx]

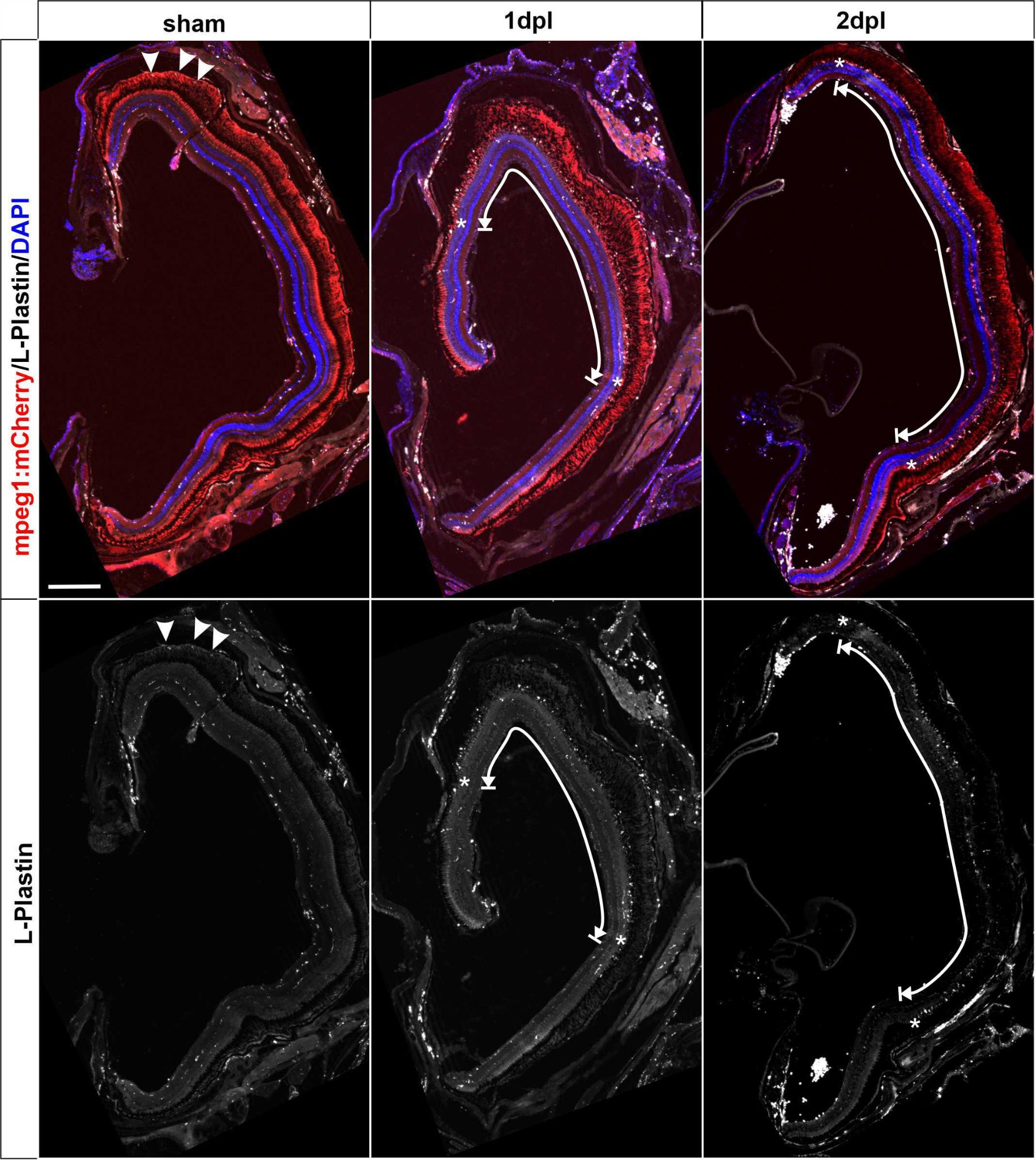


**Figure S1: Distribution of leukocytes in the retina upon acute lesion; overview.**

In low magnification cross sections of the sham control *mpeg1:mCherry*+/L-Plastin+ microglia are predominantly located in the plexiform layers of the retina; only few are detected within the rod outer segments near the RPE/choroid (arrowhead). At 1 and 2 days post light lesion (dpl), leukocytes are predominately detected at the site of the photoreceptor lesion (line with arrowhead) within the outer nuclear layer (asterisks). Scale bar = 200µm.

**
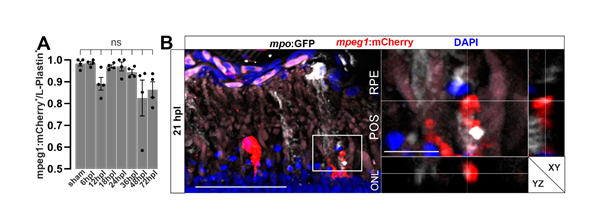
**

**Figure S2: Neutrophils as a part of *Tg(mpeg1:mCherry)-*negative leukocytes are attracted by lesion.**

(A) Quantification of *Tg*(*mpeg1*:mCherry) and L-Plastin double positive cells in the context of lesion. The amount of double positive cells decreases in response to lesion, however not significantly.

(B) *Tg*(*mpo*:GFP)^+^ neutrophils spread GFP positive matrices at the side of injury. These structures are in close proximity to *Tg(mpeg1:mCherry)* positive monocytes.

Scale bars = 50µm; Insets = 10µm; Error bars indicate SEM; ns>0.05; ** = p≤0,01; *** = p<0,001; N=4; one-way ANOVA. ONL=outer nuclear layer; GCL=ganglion cell layer, RPE= retina pigment epithelium, POS=photoreceptor outer segments.


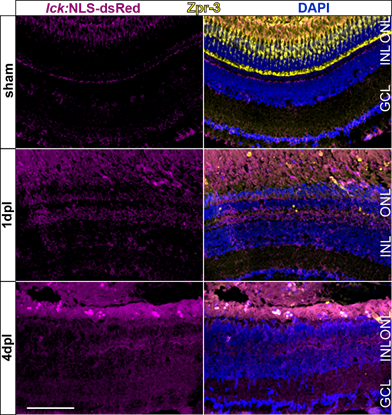


**Figure S3: *Tg(lck:NLS-dsRed)^+^* T cells do not respond to retinal injury.**

No *Tg(lck:NLS-dsRed)* T cell is detected upon injury. Photoreceptor ablation is indicated by the lack of Zpr-3 positive rods at 1 and 4 days post lesion (dpl). Scale bar = 50µm. ONL=outer nuclear layer; INL=inner nuclear layer; GCL=ganglion cell layer.


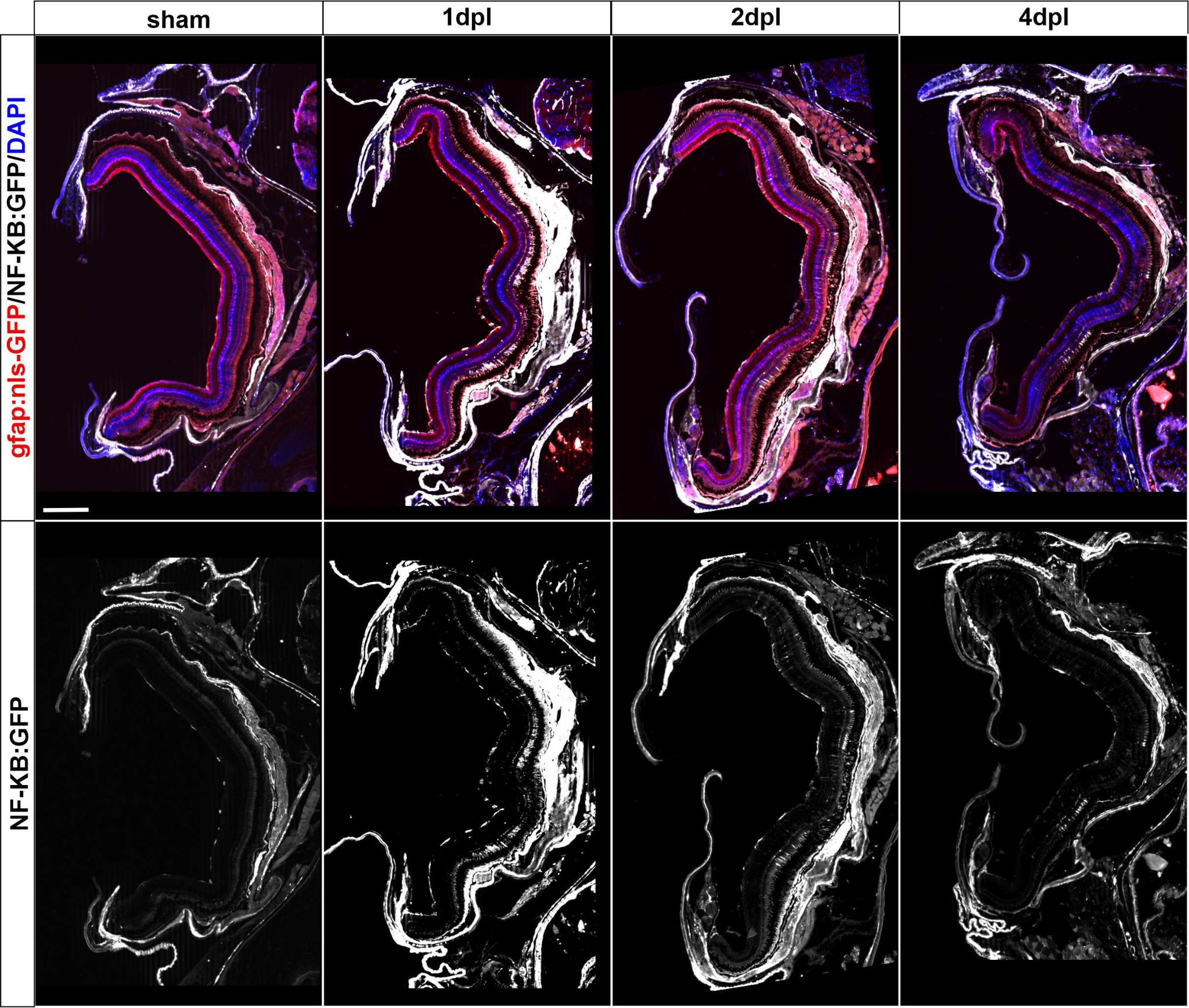


**Figure S4: NF-κB activity in whole retinal cross sections.**

In the homeostatic retina (sham), NF-κB activity is visible in blood vessels and in various structures surrounding the neuronal retina. Upon lesion, transcriptional activity of NF-κB is apparent in Müller cells (co-expressing *gfap*:nls-GFP; for display in red) and photoreceptors at the site of light lesion. The signal decreases within 4 days post lesion (dpl) but remains present in some cells. Scale bar = 200µm.


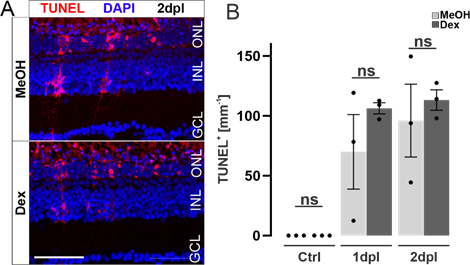


**Figure S5: Dexamethasone treatment has no influence on cell death induced by phototoxic ablation**.

(A) TUNEL^+^ nuclei are detectable in the outer nuclear Layer (ONL) of Dex and MeOH treated samples.

(B) Quantification of TUNEL^+^ cells in sham, 1 dpl and 2 dpl retina in Dex-treated eyes in comparison to vehicle control show no difference.

Scale bar = 50 µm, Error bars indicate SEM; ns = p > 0,05; N=3; two-tailed T-Test; INL=inner nuclear layer; GCL=ganglion cell layer.


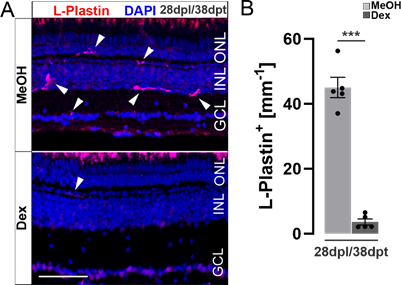


**Figure S6: Long-term dexamethasone treatment leads to a strong reduction of retinal leukocytes.**

(A) After 38 days of continuous Dex-treatment/28 dpl L-Plastin^+^ cells can rarely be detected in Dex samples in comparison to MeOH control.

(B) Quantification of L-Plastin^+^ cells in the retina of MeOH and Dex-treated fish reveals a strong decrease of L-Plastin^+^ cells.

Scale bar = 50 µm, Error bars indicate standard error; *** = p < 0,001; N=5; two-tailed T-Test; ONL=outer nuclear layer; INL=inner nuclear layer; GCL=ganglion cell layer.


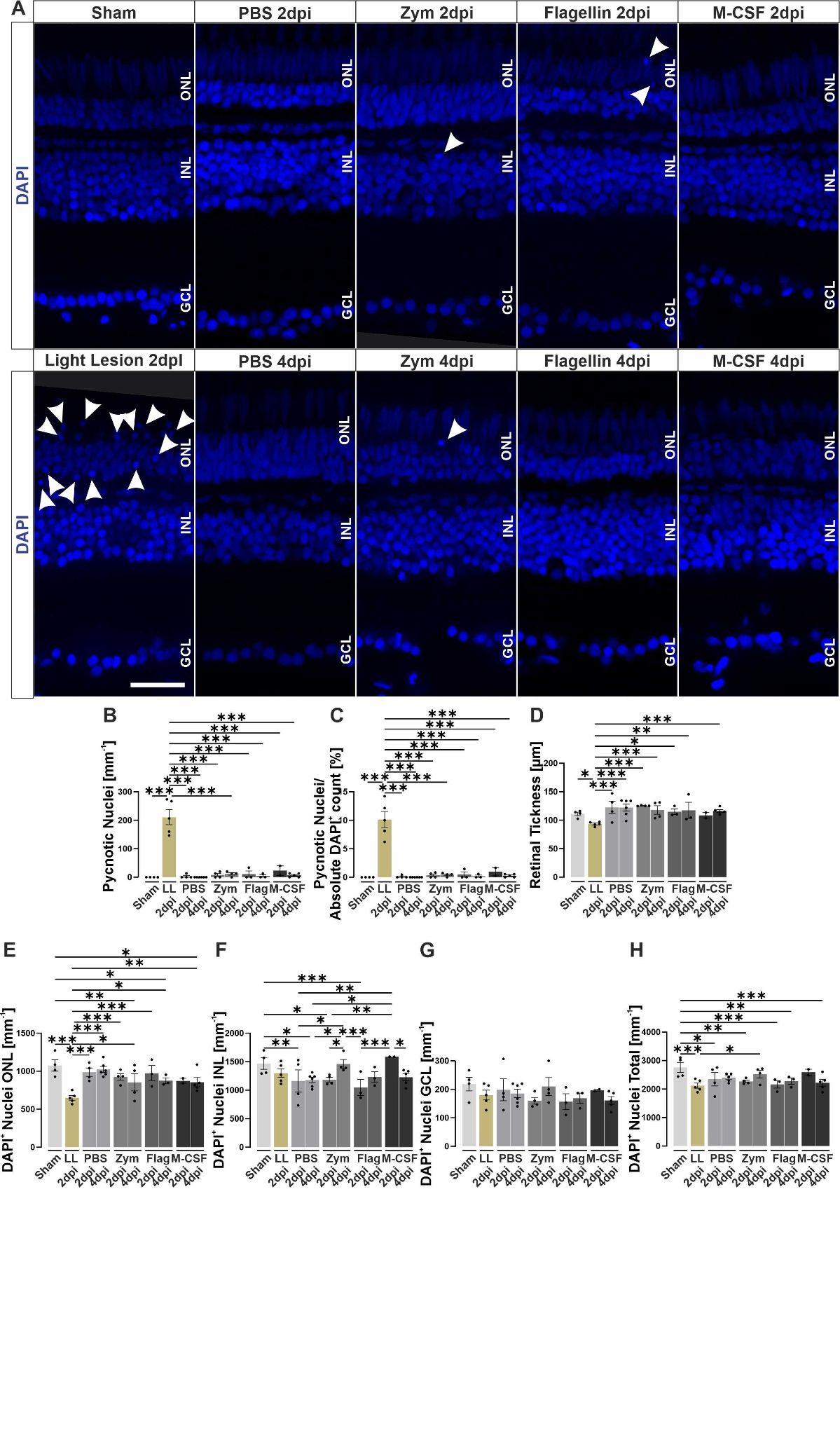


**Figure S7: Pyknotic nuclei upon injection of inflammatory stimuli.**

(A) DAPI staining highlights the nuclear architecture of the retina in different conditions. In sham no pyknotic nuclei are detected, while at 2 days post light lesion an increased amount is visible in the outer nuclear layer (ONL, arrowheads). In the injected eyes, pyknotic nuclei are only occasionally detected (arrowheads).

(B & C) Quantification of pyknotic nuclei with respect to retinal length (B) or the total amount of nuclei show strong retinal damage after the light lesion, but only sparse detection among the injected groups.

(D) The total retinal thickness is decreased in the light lesioned condition, but appears similar between injected and sham conditions.

(E, F, G & H) Quantification of DAPI positive nuclei with respect to the retinal layers indicate a local decline of cells in the ONL of light lesioned fish. The injected eyes show a heterogeneous amount of nuclei in the various layers; the overall number of retinal nuclei (H) showed no effect in comparison to the PBS injected control.

Scale bar = 25 µm. PBS=Phosphate buffered saline; Zym=Zymosan; Fla=Flagellin; LL=light lesion. Error bars indicate standard error; * = p≤0,05; ** = p≤0,01; *** = p<0,001; N=2-5 fish; ANOVA. ONL=outer nuclear layer; INL=inner nuclear layer; GCL=ganglion cell layer.
